# UNC50 Prompts G1/S Transition and Proliferation in HCC by Regulation of Epidermal Growth Factor Receptor Trafficking

**DOI:** 10.1371/journal.pone.0119338

**Published:** 2015-03-04

**Authors:** Zhou Fang, Linuo Zhou, Songmin Jiang, Lihuan Cao, Long Yu

**Affiliations:** 1 The State Key Laboratory of Genetic Engineering, School of Life Sciences, Fudan University, Shanghai, P. R. China; 2 Department of Endocrinology and Metabolism, Huashan Hospital of Fudan University, Shanghai, P. R. China; 3 Institute of Biomedical Sciences, Fudan University, Shanghai, P. R. China; University of Hong Kong, HONG KONG

## Abstract

**Background:**

UNC50 has long been recognized as a Golgi apparatus protein in yeast, and is involved in nicotinic receptor trafficking in *Caenorhabditis elegans*, but little is known about *UNC50* gene function in human biology despite it being conserved from yeast to high eukaryotes.

**Objectives:**

We investigated the relation between UNC50 and human hepatocellular carcinoma (HCC) and the potential mechanisms underlying HCC development.

**Methods:**

*UNC50* mRNA expression patterns in 12 HCC and adjacent non-cancerous tissues determined using northern blotting were confirmed by real-time PCR in another 44 paired tissues. Microarray experiments were used to screen for global effects of UNC50 knockdown in the Hep3B cell line, and were confirmed by real-time PCR, western blotting, flow cytometry, and tetrazolium assay in both UNC50 overexpression and knockdown Hep3B cells.

**Results:**

UNC50 expression levels were upregulated in HCC tissues in comparison with the adjacent non-cancerous tissues. UNC50 knockdown reduced mRNA levels of the downstream targets of the epidermal growth factor receptor (EGFR) pathway: cyclin D1 (*CCND1*), *EGF*, matrix metalloproteinase-7 (*MMP7*), aldose reductase-like 1 (*AKR1B10*), cell surface–associated mucin 1 (*MUC1*), and gastrin (*GAST*). Moreover, UNC50 influenced EGF, inducing cell cycle entry by affecting cell surface EGFR amounts.

**Conclusions:**

*UNC50* may plays some roles in HCC progression by affecting the EGFR pathway.

## Introduction

Hepatocellular carcinoma (HCC) is one of the most malignant cancers worldwide, accounting for millions of deaths every year [1]. Although HCC has been intensively studied, the molecular basis underlying HCC progression remains largely elusive. DNA microarrays have aided in the discovery of novel genes that are differentially expressed in HCC in comparison to non-cancerous adjacent tissues [2]. However, many of these genes have not been investigated closely, and their functions in humans are largely unknown. Such genes should be characterized to uncover novel tumor markers, oncogenes, and therapeutic targets for HCC. To obtain this information, we must find the crosstalk between these genes and well-known pathways.

The overexpression of receptor tyrosine kinases (RTK) is a hallmark of many cancers with poor prognosis [3]. Epidermal growth factor receptor (EGFR) is the earliest known RTK family protein, and is overexpressed in a wide range of cancers, playing important roles in cell growth and survival [4]. Specific EGFR inhibitors, such as erlotinib, gefitinib, and the monoclonal antibody cetuximab are effective for treating cancer [5]. Unfortunately, the tumor cells of most HCC patients have developed other mechanisms to bypass the EGFR pathway [6], and erlotinib is inefficient for disease control in the majority of patients with advanced HCC [7, 8]. Even so, both gefitinib and erlotinib prevented HCC development in different animal models of cirrhosis; thus, the EGFR pathway is a promising target for HCC prevention [9, 10].

UNC50 is conserved in nearly all eukaryotic organisms and is located mainly in the Golgi apparatus membrane [11]. The genes abbreviated to “UNC” followed by a numeral were first discovered during screening for mutants with the same uncoordinated motor behavior phenotype in *Caenorhabditis elegans*, and were thus named. The protein products of these genes usually have no amino acid sequence or protein structure similarity. In *C*. *elegans*, mutants of the *UNC50* gene are resistant to the acetylcholine receptor (AChR) agonist levamisole [12, 13]. Moreover, it has been demonstrated that *UNC50* plays a role in levamisole-sensitive nicotinic AChR (levi-AChR) rafficking [14] in *C*. *elegans*, indicating its crucial role in nerve signal transmission at neuron–muscle junctions. However, homologs for the *UNC50* gene are conserved in most eukaryotic organisms, including yeast and plants, which do not express AChRs; in humans, UNC50 is ubiquitously expressed in cells outside the nervous system, suggesting that the *UNC50* gene plays wider roles.

Our group first cloned and submitted the full-length sequence of human *UNC50* mRNA to the National Center for Biotechnology Information database (GeneBank ID: AY017215.1 in December 2000). In this study, we illustrate that UNC50 is overexpressed in HCC, and aim to uncover one of the roles UNC50 plays in HCC progression and the potential underlying molecular mechanisms.

## Materials and Methods

### Tissue specimen collection

Fresh surgical specimens of HCC, which comprised tumor tissues and adjacent non-cancerous liver tissues, were obtained from 56 HCC patients at Zhongshan Hospital, Shanghai, China. All samples were immediately frozen in liquid nitrogen after surgery and then stored at -80°C for further analysis.

### Ethics statement

Prior to surgery, patients signed written informed consent forms for surgery and were presented the following option: “I agree to donate my resected tissue samples to Zhongshan Hospital and relevant research groups for research.” The tissue samples of those who had selected this option were stored for research purposes. The written informed consent forms for surgery were stored by Zhongshan Hospital. In addition, verbal informed consent was obtained and recorded by our research group from all patients during our telephone follow-up. The present study was approved by the Ethics Committee of the Fudan University, Shanghai, China.

### Plasmid construction

To construct the mammalian expression vector pcDNA3.1-B(-)-UNC50, the full-length open reading frame of UNC50 according to NM_014044.5 was cloned using PCR into pcDNA3.1-B(-).

We constructed pLKO.1-shRNA-MOCK (shR-MOCK), and pLKO.1-shRNA-UNC50-554 (shR-554) and pLKO.1-shRNA-UNC50 (shR-749), containing a mock control small hairpin RNA (shRNA) sequence and a 21-nucleotide target UNC50 shRNA sequence, respectively, cloned into pLKO.1 plasmids to generate UNC50 knockdown cell lines. The detailed sequence and cloning strategy for pLKO.1 can be found at the Public TRC Portal of the Broad Institute of MIT and Harvard (http://www.broadinstitute.org/rnai/public/).

### Cell culture and transfection

The human HCC cell line Hep3B was purchased from ATCC and cultured in Dulbecco’s modified Eagle’s medium (DMEM) containing 10% fetal bovine serum (FBS; HyClone) in a humidified incubator (5% CO_2_) at 37°C.

Plasmids were transfected into Hep3B cells with Lipofectamine 2000 (Invitrogen) according to the manufacturer’s instructions. Puromycin (1 μg/ml) was added to the cells for two days to purify UNC50 knockdown cells; 200 μg/ml G418 was added to the cells for two weeks to purify UNC50 overexpression cells.

### Cell cycle analysis

Cultured cells were trypsinized, washed with phosphate-buffered saline (PBS) twice, and then permeabilized with 0.05% Triton X-100 in PBS for 10 minutes. The permeabilized cells were stained with 10 μg/ml propidium iodide solution for 10 minutes in a dark box. The treated cells were analyzed by flow cytometry (FACSCalibur; BD Bioscience) to determine DNA content and cell cycle status. We collected 10,000 gated cells for each sample.

### RNA preparation, reverse transcription, and quantitative real-time PCR

Total RNA was extracted from tissues or cultured cells using TRIzol (Invitrogen) according the manufacturer’s protocol. RNA (1 μg) was used for reverse transcription with ReverTra Ace (Toyobo) using random hexameric oligos at 42°C for 1 hour, and the complementary DNA products were appropriately diluted with Milli-Q water. To rule out contamination, additional reverse transcription reactions containing no RNA were prepared as the negative controls for real-time PCR. Quantitative real-time PCR was carried out using a SYBR qPCR Mix (Toyobo) in a 10-μl reaction volume with a LightCycler 480 II (Roche) in 384-well plates (Axygen). Each reaction was repeated in three wells. We confirmed the specificity of the real-time PCR products by melting curve analysis. All gene expression levels were normalized to β-actin.

### Microarray analysis

We analyzed total RNA from UNC50 knockdown and control Hep3B cells with Agilent Gene Expression oligo microarrays. The results have been uploaded to the Gene Expression Omnibus (GEO) database (GEO accession number: GSE63322). Genes with an average negative or positive fold change of ≥1.5 times were analyzed further. Datasets were taxonomized with FunNet (http://www.funnet.info/). Gene interaction networks were drawn using STRING (http://string-db.org/).

### Northern blotting

Total tissue RNA (10 μg) was separated on 1.5% agarose gels containing 2.2 M formaldehyde and transferred onto Hybond XL membranes. The membranes were probed with α-32P-UTP (800 Ci/mmol; PerkinElmer)-labeled minus or plus strand–specific full-length UNC-50 riboprobes and exposed to a phosphorimager screen.

### Western blotting

Cells were lysed in sodium dodecyl sulfate–polyacrylamide gel electrophoresis (SDS-PAGE) loading buffer and boiled in water for 10 minutes. Approximately 20 μg total protein extracts were loaded and separated by SDS-PAGE, and then transferred onto nitrocellulose membranes (Millipore). The membranes were blocked with 5% non-fat milk in Tris-buffered saline for 1 hour at room temperature, and then incubated with specific antibodies against EGFR, phosphorylated EGFR (pEGFR), cyclin D1 (CCND1) (Cell Signaling Technology), β-actin, myc-epitope (Sigma-Aldrich), and UNC50 (Abgent) at the appropriate dilutions at 4°C overnight. This was followed by incubation with a corresponding horseradish peroxidase–conjugated secondary antibody (Santa Cruz Biotechnology) at room temperature for 1 hour. The membrane was immersed in chemiluminescence reagent (7SeaPharmTech) and exposed with G:BOX (Syngene).

### Tetrazolium assay

Cells (1000 cells per well) were seeded onto 96-well plates (Falcon) and underwent tetrazolium (MTT) assay (Dojindo) every day. The absorbance of each well was measured 1 hour after incubation using a microtiter reader (Bio-Rad) at 530 nm.

### Flow cytometry

Cultured cells (1 × 10^6^) from each sample were trypsinized, resuspended in PBS, and fixed in 4% formaldehyde for 10 minutes at room temperature. Cells from each sample were divided into two portions for either extracellular or whole-cell staining. For the latter, cells were additionally permeabilized in chilled 90% methanol for 30 minutes on ice. Both portions were then incubated in 0.5% bovine serum albumin/PBS solution for 5 minutes twice. Subsequently, the cells were resuspended for 1 hour at room temperature away from light in 100 μl 1:10 diluted Alexa Fluor 488–conjugated EGFR antibody (Santa Cruz Biotechnology) recognizing the cell surface epitope of EGFR. The cells were washed by centrifugation in PBS twice, resuspended, and analyzed using a flow cytometer (FACSCalibur; BD Biosciences). We collected 10,000 gated cells from each sample.

### Statistical analysis

Stata 10 statistical software (StataCorp LP) was used for statistical analysis. All data are expressed as the mean ± standard error of the mean. Statistical analysis of the data was performed using Student’s *t*-test. P < 0.05 was considered significant. Materials and methods for meta-analyses have been included in S1 Text.

## Results

### UNC50 was upregulated in HCC

**Fig 1:**
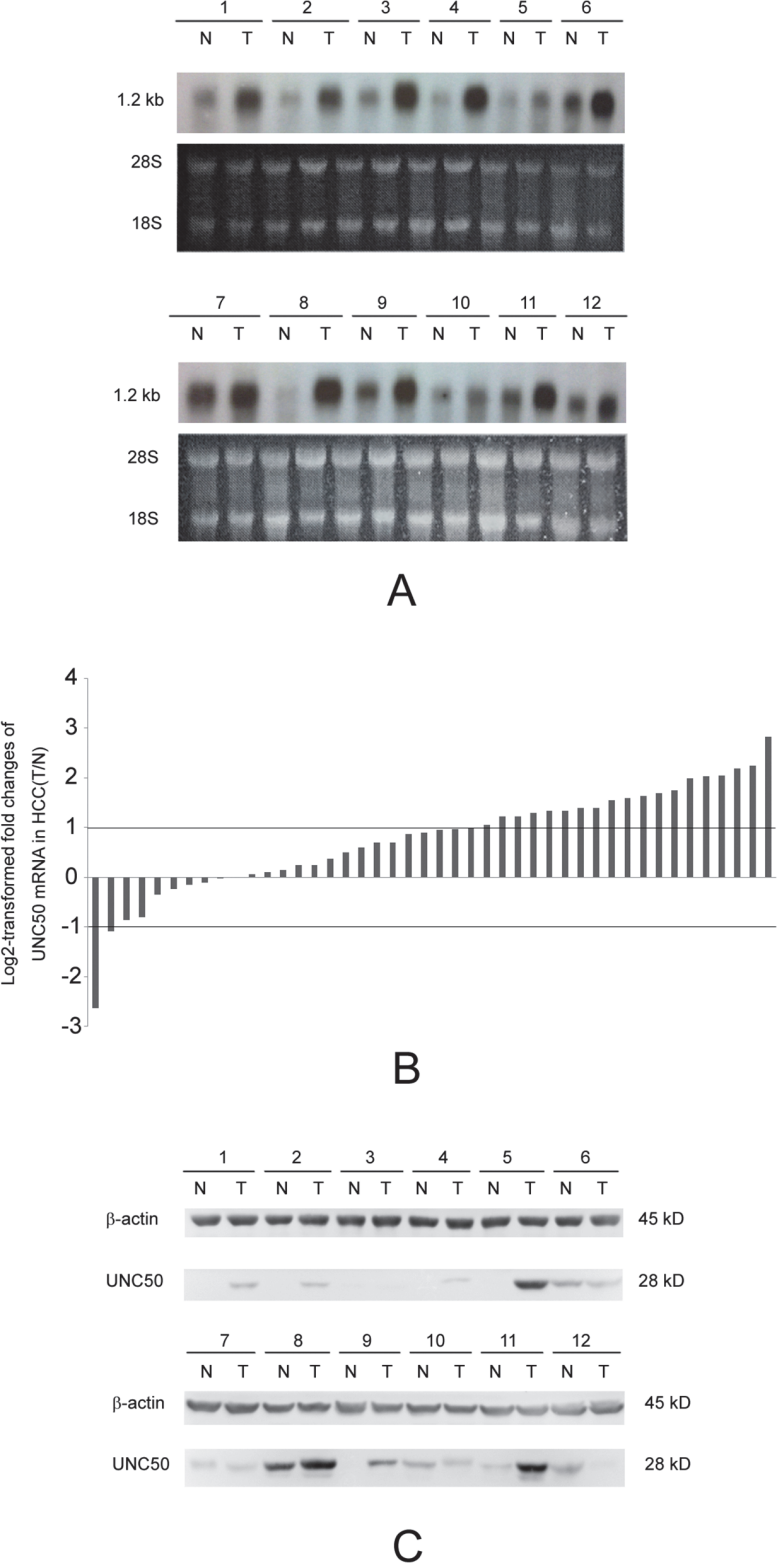
Increased UNC50 expression in HCC. (A) Northern blotting analysis of *UNC50* mRNA expression levels in 12 paired HCC tissues (T) and adjacent non-cancerous tissues (N); 28S and 18S bands were used as the references. (B) Real-time PCR analysis of *UNC50* expression levels in 44 paired tissues. Relative mRNA expression levels are normalized to β-actin; log2-transformed fold changes of HCC tissues compared to the adjacent non-cancerous tissues were calculated by the comparative cycle threshold (ΔΔCt) method. The paired tissues are ordered from low to high ΔΔCt values. Cutoff values were set to ±1 to identify the significance of changes. (C) Western blotting analysis of UNC50 levels in another 12 paired HCC tissues; β-actin was used as the reference.

We identified *UNC50* as a potential upregulated gene in human HCC compared to the adjacent non-cancerous tissues during our initial screening for HCC-relevant genes by northern blotting (Fig. 1A). Quantitative PCR of *UNC50* mRNA expression levels in the 44 paired HCC samples confirmed this. Overall, 20 of 44 (45.5%) HCC cases showed significant UNC50 upregulation, 22 of 44 (50%) showed no alteration, and only two of 44 (4.5%) showed reduced UNC50 (Fig. 1B). In agreement with this, western blotting revealed that UNC50 was detectable in eleven of 12 cancer tissues and in only six of 12 non-cancerous tissues (Fig. 1C). Following systematic review of 16 independent microarray experiments in the GEO database, our meta-analysis further showed that UNC50 was significantly upregulated in HCC tissues in comparison with the paired adjacent non-cancerous liver tissues (p = 0.005) (S1 Fig.). Begg’s funnel plot was used to illustrate publication bias (S1 Fig.). The studies used in the meta-analysis have been included in S1 Table.

### The influence of UNC50 knockdown on gene expression patterns

**Fig 2:**
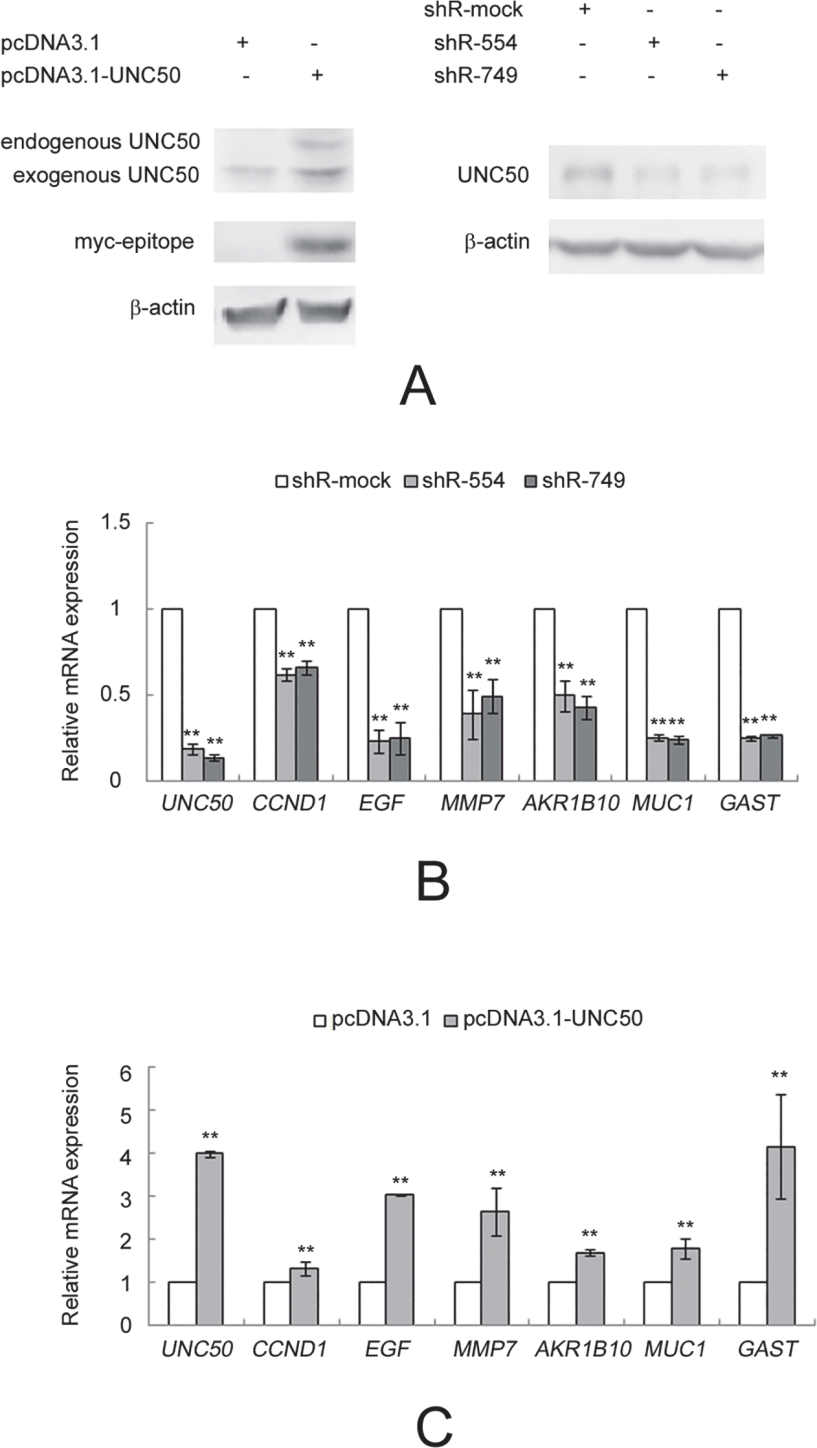
Confirmation of microarray results in UNC50 overexpression and knockdown Hep3B cells. (A) Western blotting confirmation of the effectiveness of UNC50 overexpression and knockdown; β-actin served as the reference. (B, C) Real-time PCR confirmation of seven regulated genes in response to UNC50 (B) knockdown or (C) overexpression; expression levels were normalized against β-actin. **: p<0.01.

To gain insight into the role UNC50 plays in HCC progression, we used microarray analyses to identify indirect evidence of *UNC50* gene function via the knockdown strategy in Hep3B cells. Hep3B cells transfected with the shRNA expression plasmids shR-554, shR-749, and shR-MOCK were purified with 1 μg/ml puromycin, and the total RNA from each cell was extracted and analyzed with oligo microarrays. Fig. 2A shows that *UNC50* knockdown or overexpression successfully altered UNC50 levels in comparison with the control. Following statistical selection of the regulated transcripts in the group, we found that 94 genes were regulated. Among them, several altered genes are the downstream targets of the EGFR pathway, including *CCND1*, *EGF*, matrix metalloproteinase-7 (*MMP7*) [15], aldose reductase-like 1 (*AKR1B10*) [16], cell surface–associated mucin 1 (*MUC1*) [17], and gastrin (*GAST*) [18]. Table 1 lists the microarray data. The microarray results were confirmed by real-time PCR analysis of *UNC50*, *CCND1*, *EGF*, *MMP7*, *AKR1B10*, *MUC1*, and *GAST* (Fig. 2B) in UNC50 overexpression and knockdown Hep3B cells.

**Table 1:** List of main regulated genes downstream of the EGFR pathway in UNC50 knockdown Hep3B cells.

Gene Symbol	Probe Name	Gene Name	Fold Change (log2 transformed)		
			shR-467	shR-554	shR-749
UNC50	A_23_P252700	unc-50 homolog	-2.05	-2.90	-2.40
CCND1	A_23_P202837	cyclin D1	-1.42	-1.68	-1.47
EGF	A_23_P155979	epidermal growth Factor	-2.23	-1.55	-1.76
MMP7	A_23_P52761	matrix metallopeptidase 7	-1.72	-1.42	-1.14
AKR1B10	A_24_P129341	aldo-keto reductase family 1, member B10	-3.01	-2.74	-0.83
MUC1	A_33_P3332215	mucin 1	-1.65	-1.27	-0.98
GAST	A_23_P159191	gastrin	-1.70	-2.64	-0.67

### UNC50 activates EGFR pathway in a ligand-dependent manner

**Fig 3:**
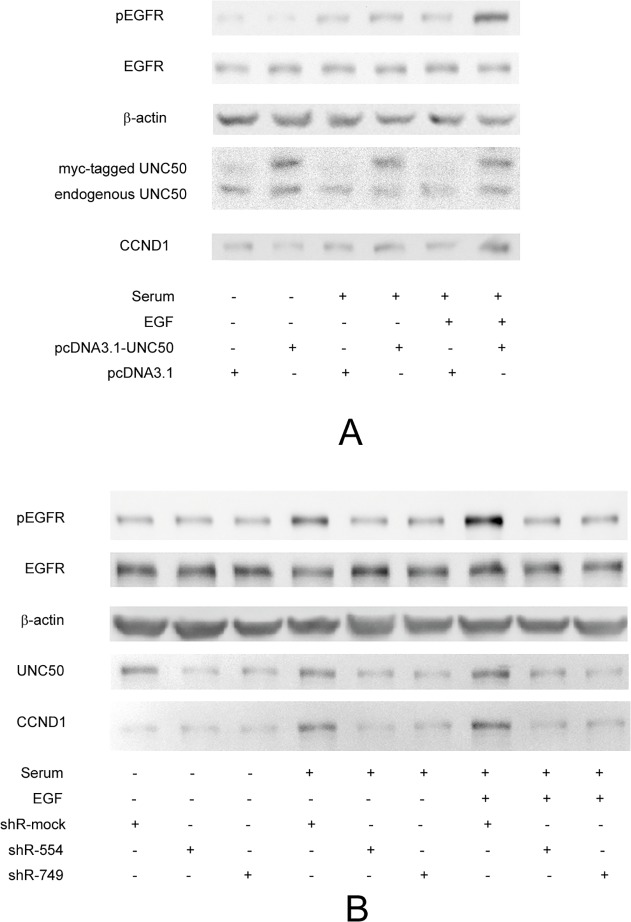
Western blotting confirmation that UNC50 affects cellular EGFR pathway activity. Cells (10^5^ from each cell type) were seeded to 24-well plates in DMEM with 10% FBS (Complete medium). After adherence, cells were pre-treated with serum free DMEM for 24 hours before being stimulated with either flesh serum free DMEM, complete medium, or complete medium with 1ng/ml EGF for 8 hours. Lysates were then collected and analyzed for pEGFR, EGFR, β-actin, cyclin D1 and UNC50 by western blot. SF = serum-free DMEM.

We assumed that UNC50 plays a role in the EGFR pathway. The EGFR pathway is crucial to cell cycle progression and proliferation. However, both total EGFR protein (Fig. 3A) and *EGFR* mRNA (data not shown) levels remained unchanged, suggesting that UNC50 acts in a post-translational manner. Therefore, we evaluated EGFR pathway activity by detecting the phosphorylation levels of EGFR at tyrosine 1068 (pEGFR-1068) with immunoblot assays. As cellular pEGFR-1068 levels are sensitive to extracellular ligand stimulation (ligand-dependent) and cell–cell contact (ligand-independent), we seeded equal numbers of cells to reach similar confluence (approximately 40%) after adherence and starved the cells for 24 hours before the different treatments. As shown in Fig. 3A, the EGFR pathway activity of the cells was proportional to that of UNC50 expression levels when cultured in DMEM with 10% FBS for 8 hours. Moreover, such effects were enhanced when 1 ng/ml EGF was added. However, we were unable to detect the pEGFR-1068 levels in cells cultured in serum-free medium, indicating that ligand-independent activation was scarce. From this, we may infer that UNC50 affects EGFR pathway activity in a ligand-dependent manner.

### UNC50 prompted cell cycle entry and proliferation through the EGFR pathway

**Fig 4:**
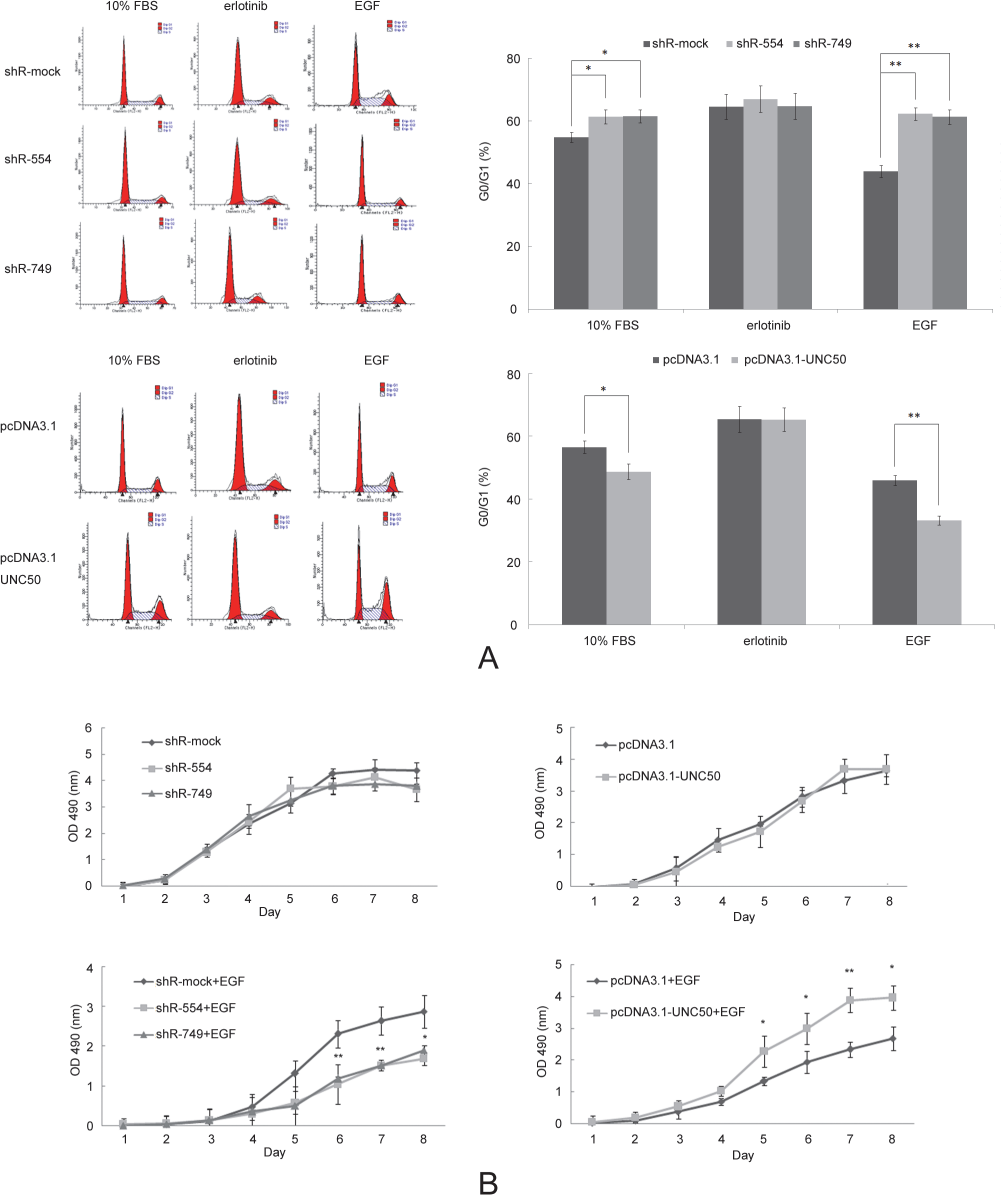
UNC50 prompts EGF-induced cell cycle entry and proliferation. (A) Flow cytometry analysis of cells stimulated with complete medium (10% FBS), complete medium with 1 ng/ml EGF (EGF) added for another 24 hours, or 10 μmol/ml erlotinib (Erlotinib) after EGF treatment. (B) MTT evaluation of cell proliferation status. Top: Cells were cultured in complete medium without change of medium; cell vitality was analyzed every 24 hours. Bottom: Cells were cultured in complete medium with 1 ng/ml EGF and replaced with fresh medium every 12 hours to sustain EGF levels. Cell vitality was analyzed every 24 hours. *: p<0.05, **: p<0.01.

CCND1 is a key component of the G1/S checkpoint and is a downstream target of the EGFR pathway. As both CCND1 mRNA (Fig. 2B) and protein levels (Fig. 3) were altered in accordance with UNC50 levels, we examined the cell cycle distribution of cells in which UNC50 expression levels had been modified. Cells were synchronized in the G0 phase through serum starvation before treatment. Following release from the G0 phase by serum stimulation, more cells were arrested in G0/G1 phase after UNC50 knockdown, and vice versa; the addition of EGF further enhanced these differences (Fig. 4A). Erlotinib, a specific EGFR inhibitor, countered the effects of EGF stimulation, indicating that the role of UNC50 in the cell cycle relies on, at least partly, a functional EGFR pathway.

To support our cell cycle results, we evaluated the cell proliferation states using the MTT assay. No significant difference was observed under normal conditions (10% FBS in DMEM). Constantly supplying low levels of EGF to the cultured cells (approximately 1 ng every 12 hours) greatly enhanced the corresponding differences among the cells, further supporting our earlier results (Fig. 4B).

### UNC50 affected cell surface EGFR amounts

**Fig 5:**
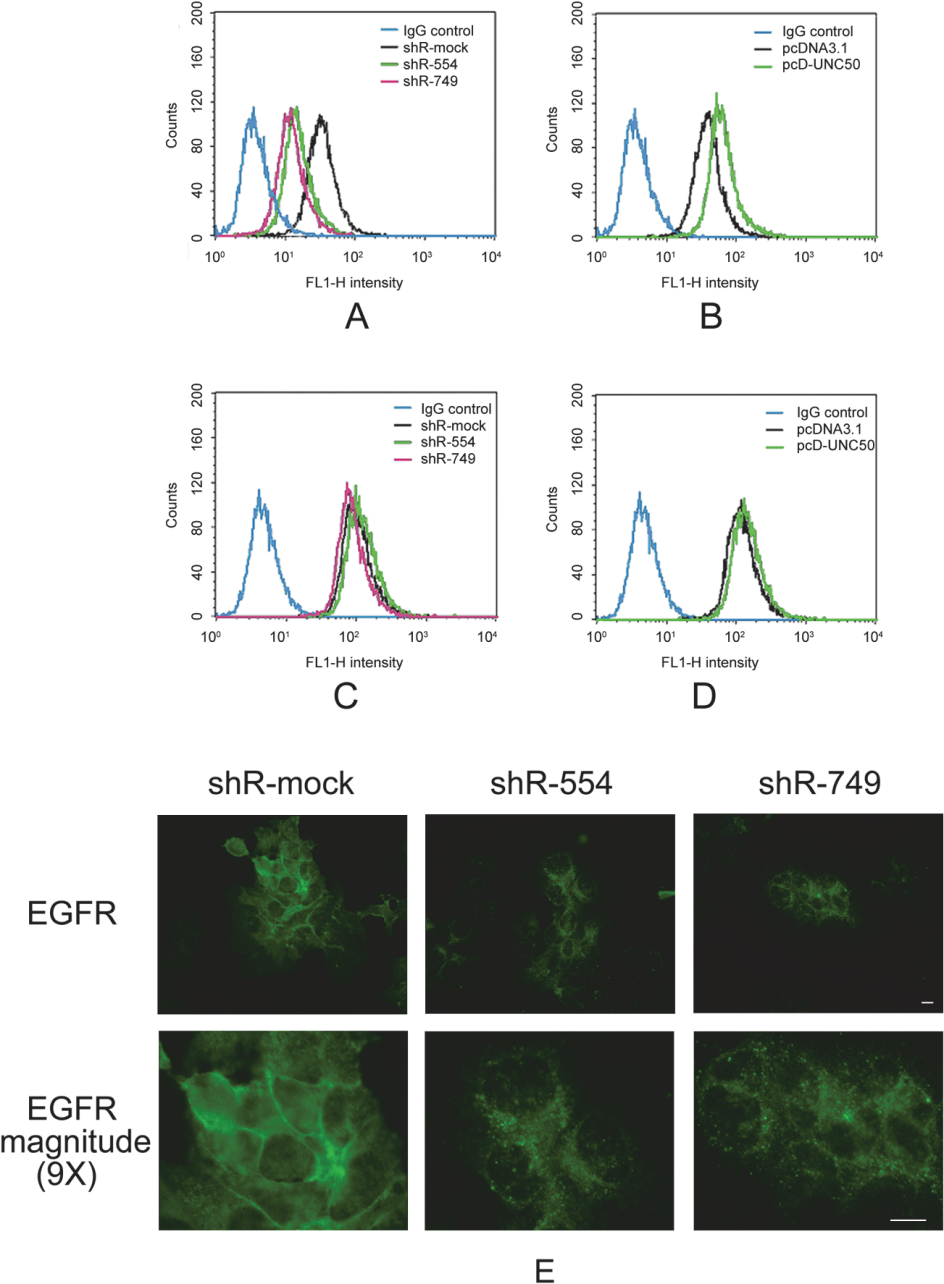
Flow cytometry and immunofluorescence analyses of EGFR distribution. (A, B) Cells not treated with 0.05% Triton X-100 before antibody staining, (C, D) Cells permeabilized by 0.05% Triton X-100 before antibody staining. Isotype rabbit immunoglobulin G (IgG) was used as the negative control. (E) Cells were stained for EGFR with Alexa 488-conjugated EGFR antibody and visualized under a fluorescence microscope at 488 nm excitation light. Scale bar is 10 μm.

As UNC50 regulates nicotinic AChR trafficking to the cell membrane [14], we hypothesized that UNC50 acts similarly with EGFR. To test this, we stained cells with fluorescence-labeled EGFR antibodies with or without cell membrane permeabilization and compared cell surface EGFR amounts using flow cytometry. To eliminate the influence of ligands on EGFR translocation, we cultured the cells in serum-free medium for 24 hours before staining. As expected, the fluorescence intensity of the non-permeabilized stained cells was weaker and proportional to UNC50 expression levels (Fig. 5A, B); the fluorescence intensity of the permeabilized cells was stronger and not correlated with UNC50 expression levels (Fig. 5C, D). Moreover, the immunofluorescence experiment showed that UNC50 knockdown dramatically altered EGFR location (Fig. 5E). We could not visualize the EGFR changes by immunofluorescence following UNC50 overexpression (Fig. not shown) because the effects of UNC50 overexpression (Fig. 5B, D) on EGFR translocation were much weaker than that of UNC50 knockdown (Fig. 5A, C). These data show that UNC50 may be crucial for EGFR distribution without much change in total cellular EGFR.

## Discussion

To our knowledge, this is the first study on UNC50 function in HCC and the second report on UNC50 in humans. The first study of the *UNC50* gene in humans illustrated the role UNC50 may play in mechanical stress in periodontal tissues [19].

In this study, we found that UNC50 is upregulated in HCC. The expression levels of many genes are dramatically altered in HCC, but we are ignorant of their functions therein. Uncovering their roles in HCC may help us understand the molecular basis underlying HCC and thus provide novel therapy targets for HCC prevention and/or therapy, and *UNC50* might be one such target.

It has been reported that UNC50 is a Golgi apparatus membrane protein [11]. Very few Golgi apparatus–related proteins are reported as being related to HCC progression despite the Golgi apparatus being a crucial cellular apparatus. GP73, a Golgi protein secreted into the serum, is a promising serum marker for diagnosing HCC [20].

Using 2D–SDS-PAGE, Yang et al. screened for Golgi proteins up- or downregulated in HCC [21]. They did not pick up the UNC50 protein spot in the matrix-assisted laser desorption/ionization–time-of-flight mass spectrometry. However, among the 17 differentially expressed proteins selected, COG8 (one of the eight subunits of the conserved oligomeric Golgi protein complex), has a yeast homolog, Cog8p, that interacts with Gmh1p (the yeast homolog for UNC50), as demonstrated in two independent large-scale screening experiments for protein interactions in yeast [22, 23]. Table 2 contains the full list of Gmh1p interaction partners.

**Table 2:** Gmh1p interaction partners in yeast.

Pubmed ID	Gene name
12808035	Arf1 Gea1** Gea2**
20093466	Arl1 Imd4 Cog5 Cog6* Cog7 Cog8* Dep1 Gdh2 Rgp1 Rml2 Rps29A Rud3* Snf2 Ypt6**
11743162	Bzz1 Lsb3 Sla1 Ysc84
23891562	Cog6* Cog8* Get2 Rud3* Sys1 Ubx2 Ypt6** Get2
22579291	Hda1
22282571	Hog1 Ptk2 Slt2 Alk2
19840948	Nab2
12436259	Pol31
14764870	Ric1 Ypt6**
23793018	Ubi4
10688190	Ypt31

*Interactions were evident in two independent experiments.

**Interactions were evident in at least three independent experiments.

Despite these findings, current knowledge of Golgi apparatus proteins in HCC progression is scanty. Our research brings a new Golgi apparatus protein, UNC50, to more attention.

Using gene expression microarray analyses, we were able to screen for the oncological roles of UNC50 indirectly by interfering with UNC50 expression levels in Hep3B cells. The Hep3B cell line lacks the retinoblastoma (*RB*) and *P53* genes, and has a hepatitis B virus (HBV) infection background. In our opinion, it is the appropriate cell model for Chinese HCC patients because most of these patients have a history of chronic HBV infection. The gene expression patterns revealed impaired expression levels of downstream targets of the EGFR pathway. We evaluated EGFR pathway activity by examining EGFR phosphorylation; western blotting showed that pEGFR levels were related to UNC50 expression, indicating that UNC50 plays a role in the EGFR pathway. In accordance with the molecular phenotype, we observed G0/G1 arrest and retardation of proliferation following UNC50 inhibition, and vice versa, upon EGF stimulation. We observed only marginal effects of UNC50 inhibition or overexpression on the cell cycle and no effect on proliferation because the ability of FBS to activate EGFR is very limited (Fig. 3A). Adding erlotinib, a specific EGFR inhibitor, abrogated the cell cycle differences, indicating that the impact of UNC50 on the cell cycle relies on a functional EGFR pathway.

Notably, total EGFR protein and *EGFR* mRNA levels did not change with UNC50 levels, thus UNC50 must act in a post-translational manner. EGFR staining with antibodies and flow cytometry analysis with or without permeabilization demonstrated that cell surface EGFR amounts are altered upon UNC50 overexpression or inhibition (Fig. 5), indicating that UNC50 influences EGFR translocation to the cell membrane despite total EGFR remaining unchanged. EGFR is rapidly internalized into endosomes and recycled to the cell surface even in the absence of ligands; endosomal retention is a key determinant for cell surface EGFR amounts [24]. UNC50 may play a role in facilitating EGFR recycling, thus increasing cell surface EGFR amounts. It is worth noting that EGFRs cycled between the cell surface and endosomes are less likely to be degraded [25]. That is why the total EGFR amount remained unchanged even when EGFR distribution was altered.

**Fig 6:**
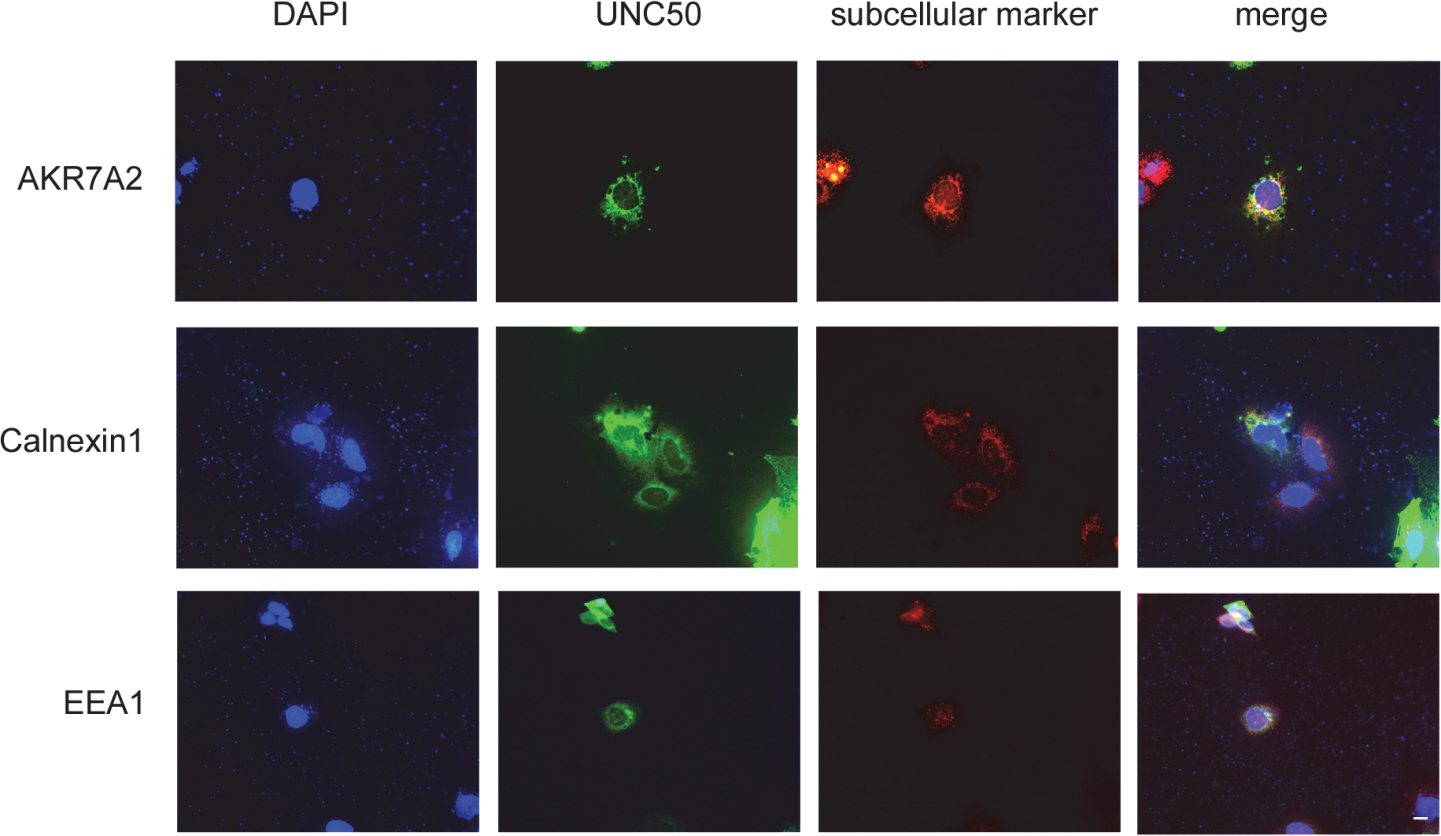
Distribution of recombinant UNC50 protein. Hep3B cells at approximately 40% confluence were transfected with 1 μg pEGFP-N1-UNC50. After 48-hour transfection, cells were fixed in 4% paraformaldehyde and stained for Golgi apparatus, endoplasmic reticulum, and early endosome markers AKR7A2, calnexin 1, and early endosomal autoantigen 1 (EEA1), respectively. Cells were examined under a fluorescence microscope at corresponding excitation light. Scale bar is 10 μm.

How could UNC50 influence the endosomal process if it is a Golgi apparatus membrane protein? We found that the recombinant UNC50 protein, with enhanced green fluorescent protein linked to its C-terminal, was located mainly in the Golgi apparatus and endoplasmic reticulum regions (Fig. 6). Moreover, an early study demonstrated that UNC50 is an inner nuclear membrane protein [26]. The Golgi apparatus is a hub for protein sorting and trafficking. A previous study showed that UNC50 affects receptor trafficking indirectly [14] and does not require colocalization with these receptors, and this might be the case for the relation between UNC50 and EGFR. The details regarding UNC50 location in HCC cells requires more evidence.

There are many types of receptors, and for UNC50 to solely affect EGFR translocation would not be logical. Eimer et al., who studied levi-AChR, encountered the same puzzle regarding the specificity of UNC50 [14]. They clarified this by assuming that UNC50 plays a redundant role in protein trafficking and that levi-AChR exclusively relies on UNC50 functions. They also mentioned that apart from impaired movement, *C*. *elegans* UNC50 mutants exhibited only marginal phenotypes, which was greatly similar to our findings. Differing from their explanation for levi-AChR, we hypothesize that UNC50 is a global factor for protein trafficking. UNC50 plays certain roles for a specific protein (not confined to the receptor), and affects the counterpart of this protein, thus its effects are neutralized. Moreover, the effects of UNC50 on a small number of proteins are not totally neutralized; as the present study reveals, EGFR happens to be one of them. This may better explain the fact that UNC50 is conserved among all eukaryotic organisms, as both EGFR and AChR do not exist in all eukaryotic organisms. However, the implications of UNC50 in other protein trafficking processes require further extensive study.

In summary, UNC50 enhances the EGFR pathway by facilitating EGFR translocation to the cell surface in the HCC cell line Hep3B, and promotes cell cycle entry and proliferation in the presence of EGF. This study relates the novel Golgi apparatus membrane protein UNC50 to an important tumor-promoting pathway that involves EGF. Further studies will determine its roles in HCC progression in detail.

## Supporting Information

S1 Fig Meta-analysis of UNC50 expression in paired HCC tissues. (A) Forest plot of UNC50 expression fold changes (log2 scale) and corresponding 95% confidence intervals. A random effects model was used to exclude the influence of heterogeneity. (B) Funnel plot depicting publication bias at 95% confidence intervals.
(TIF)

S1 Table Basic characteristics of the studies included for meta-analysis. (DOCX)

S1 Text Materials and methods for meta-analyses. (DOCX)
