# Supplementary figures and images for: UNC50 Prompts G1/S Transition and Proliferation in HCC by Regulation of Epidermal Growth Factor Receptor Trafficking
Source: PLoS One. 2015 Mar 4;10(3):e0119338. doi: 10.1371/journal.pone.0119338 (PMC4349650; doi:10.1371/journal.pone.0119338)

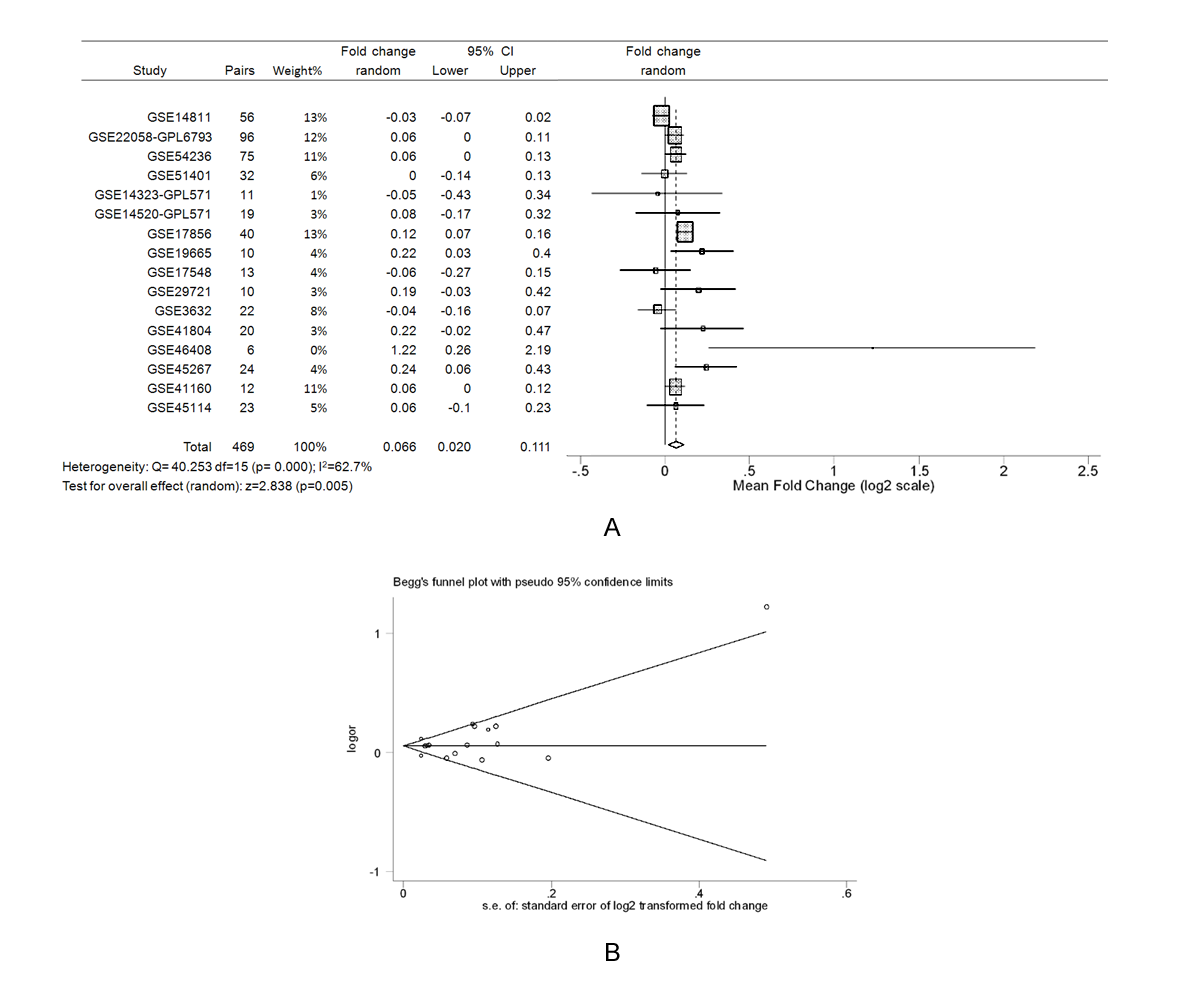

Supplement: S1 Fig — (A) Forest plot of UNC50 expression fold changes (log2 scale) and corresponding 95% confidence intervals. A random effects model was used to exclude the influence of heterogeneity. (B) Funnel plot depicting publication bias at 95% confidence intervals. (TIF) [file pone.0119338.s001.tif]
