# Supplementary material for: UNC50 Prompts G1/S Transition and Proliferation in HCC by Regulation of Epidermal Growth Factor Receptor Trafficking
Source: PLoS One. 2015 Mar 4;10(3):e0119338. doi: 10.1371/journal.pone.0119338 (PMC4349650; doi:10.1371/journal.pone.0119338)
Supplement: S1 Table — (DOCX) [file pone.0119338.s002.docx]

**S1 Table**. Basic characteristics of the studies included for meta-analysis

| GEO accession | year | country | number | platform | Channel^*^ | Contributor | contact name |
| --- | --- | --- | --- | --- | --- | --- | --- |
| GSE14811 | 2009 | South Korea | 56 | others | 2 | Kim B, et al | Kim B |
| GSE22058-GPL6793 | 2010 | Hong Kong | 96 | Merck | 1 | Burchard J, et al | Burchard J |
| GSE54236 | 2014 | Italy | 75 | Agilent | 1 | Villa E, et al | Villa E |
| GSE51401 | 2013 | China | 32 | Affymetrix | 1 | Sun H, et al | Kong LQ |
| GSE14323-GPL571 | 2009 | USA | 11 | Affymetrix | 1 | Mas VR, et al | Archer KJ |
| GSE14520-GPL571 | 2009 | USA | 19 | Affymetrix | 1 | Roessler S, et al | Wang XW |
| GSE17856 | 2009 | USA | 40 | Agilent | 2 | Tsuchiya M, et al | Rusyn I |
| GSE19665 | 2009 | Japan | 10 | Affymetrix | 1 | Nagae G, et al | Kaneda A |
| GSE17548 | 2009 | Turkey | 13 | Affymetrix | 1 | Ozturk M, et al | Ozturk M |
| GSE29721 | 2011 | Canada | 10 | Affymetrix | 1 | Bhattacharyya B, et al | Suderman M |
| GSE3632 | 2005 | France | 22 | others | 1 | Derambure C, et al | Salier J |
| GSE41804 | 2012 | Japan | 20 | Affymetrix | 1 | Hodo Y, et al | Hodo Y |
| GSE46408 | 2013 | Taiwan | 6 | Agilent | 1 | Jeng Y, et al | Jeng Y |
| GSE45267 | 2013 | Taiwan | 24 | Affymetrix | 1 | Wang H, et al | Hsieh JY |
| GSE41160 | 2012 | France | 12 | Illumina | 1 | Caboux E, et al | Calvez-kelm F |
| GSE45114 | 2013 | China | 23 | others | 2 | Wei L, et al | Wei L |

*Microarrays use only one channel (cyanine [Cy] 3 or Cy5) or two channels (both Cy3 and Cy5)
