# Supplementary material for: UNC50 Prompts G1/S Transition and Proliferation in HCC by Regulation of Epidermal Growth Factor Receptor Trafficking
Source: PLoS One. 2015 Mar 4;10(3):e0119338. doi: 10.1371/journal.pone.0119338 (PMC4349650; doi:10.1371/journal.pone.0119338)
Supplement: S1 Text — (DOCX) [file pone.0119338.s003.docx]

**Materials and Methods**

*Data collection*

Using the key words “hepatocellular carcinoma” and filters “Series” (entry type), “*Homo* *sapiens*” (organism), “expression profiling by array” or “expression profiling by high throughput sequencing” (study type), and “tissue” (attribute name), we searched the GEO database for datasets uploaded before September 2010. We retrieved independent datasets containing paired HCC tissues and adjacent non-cancerous tissues. The datasets used in this study are listed in S1 Table. The normalized microarray data of the paired tissue samples were downloaded directly from the GEO database.

*Data processing*

All samples and corresponding data were paired manually according to the sample descriptions. Probe signal intensity for *UNC50* gene expression was extracted from these data. Except the microarray data with log2-transformed signal intensity, the signal intensity are log2-transformed. Log2-transformed fold change of UNC50 expression level was calculated by subtracting the UNC50 signal intensity of HCC tissues from that of the paired noncancerous tissue. Meta-analyses were performed using a random effects model, and a forest plot was drawn using STATA 12. The mean log2-transformed fold change and 95% confidence interval were calculated. Begg’s funnel plot was drawn using STATA 12 to visualize publication bias for the meta-analyses.
